# Cu-Based Conductive MOF Grown *in situ* on Cu Foam as a Highly Selective and Stable Non-Enzymatic Glucose Sensor

**DOI:** 10.3389/fchem.2021.786970

**Published:** 2021-11-29

**Authors:** Qin Hu, Jie Qin, Xiao-Feng Wang, Guang-Ying Ran, Qiang Wang, Guang-Xiang Liu, Jian-Ping Ma, Jing-Yuan Ge, Hai-Ying Wang

**Affiliations:** ^1^ College of Chemistry and Materials Science, Sichuan Normal University, Chengdu, China; ^2^ School of Life Sciences and Medicine, Shandong University of Technology, Zibo, China; ^3^ School of Environmental Science, Nanjing Xiaozhuang University, Nanjing, China; ^4^ School of Chemistry, Chemical Engineering and Materials Science, Shandong Normal University, Jinan, China; ^5^ College of Chemistry and Materials Engineering, Wenzhou University, Wenzhou, China

**Keywords:** non-enzymatic, electrochemical sensor, glucose, MOF, redox activity

## Abstract

A non-enzymatic electrochemical sensor for glucose detection is executed by using a conductive metal–organic framework (MOF) Cu-MOF, which is built from the 2,3,6,7,10,11-hexahydroxytriphenylene (HHTP) ligand and copper acetate by hydrothermal reaction. The Cu-MOF demonstrates superior electrocatalytic activity for glucose oxidation under alkaline pH conditions. As an excellent non-enzymatic sensor, the Cu-MOF grown on Cu foam (Cu-MOF/CF) displays an ultra-low detection limit of 0.076 μM through a wide concentration range (0.001–0.95 mM) and a strong sensitivity of 30,030 mA μM^−1^ cm^−2^. Overall, the Cu-MOF/CF exhibits a low detection limit, high selectivity, excellent stability, fast response time, and good practical application feasibility for glucose detection and can promote the development of MOF materials in the field of electrochemical sensors.

## Introduction

Glucose is the direct source of energy for the human body and is indispensable for human functional movement. However, abnormal glucose concentration such as hypoglycemia and diabetes can lead to death and disability (Pandey et al., 2011). Therefore, the detection of glucose is of great significance to human health. Over the past few decades, many techniques have been used to detect glucose in human blood, such as colorimetry (Liu et al., 2011), gas chromatography (Larsen, 2015), fluorescence (Xie et al., 2017), and electrochemical sensors (Lpa et al., 2019; Xu et al., 2021). Among them, the accuracy of colorimetry is poor, the gas chromatography equipment is complex and expensive (Feng et al., 2016; Hui and Ying, 2017; Wang et al., 2020), and the fluorescence is very sensitive to some interfering substances (Zheng et al., 2019). Compared with enzyme electrochemical sensors, the non-enzymatic electrochemical sensor has the advantages of long service life, high cost performance, excellent stability, simple operation, and easy to carry (Clark and Lyons, 2010; Hui et al., 2016; Hui et al., 2017; Zhang et al., 2017; Archana et al., 2019; Zhao et al., 2020). Above all, it is very necessary to develop a non-enzymatic sensor with excellent performance (Hua et al., 2016; Niu et al., 2016).

Metal–organic frameworks (MOFs) are a family of novel porous crystalline materials composed of organic linkers and inorganic metal ions/clusters and have attracted wide attention for their potential applications in many fields (Hmadeh et al., 2012; Chandran et al., 2017), such as catalysis, gas storage and separation, and energy storage and conversion (Adil et al., 2017; Doonan et al., 2017; Stassen et al., 2017; Wang et al., 2018). In recent years, MOFs and their derivatives have been considered promising candidates for electrochemical sensors due to their structure adjustability and fascinating properties, such as high surface area, flexible pore size, exposed active sites, and chemical stability (Hou et al., 2013; Wang et al., 2013). However, the low conductivity of most primitive MOFs limits their application in electrochemical sensing (Wei and Yin, 2015; Usman et al., 2016; Kanj et al., 2019). The common solutions to improve the conductivity of materials are coupling MOFs with conductive materials such as carbon black and electroconductive rubber. However, these methods not only increase the series resistance but also lead to reduction in the surface area, blocking the active sites and hindering the diffusion of target molecules (Zhu et al., 2016; Ko et al., 2018). Therefore, it still remains a huge challenge to prepare pristine MOFs as efficient electrocatalysts for glucose oxidation. Triphenylene (TP) is a planar-conjugated polycyclic aromatic hydrocarbon, and the organic ligands containing the TP unit can be used as starting material to construct novel multifunctional MOF materials and covalent organic frameworks (COFs) (Hmadeh et al., 2012). Some MOFs based on the TP ligand not only exhibit good conductivity but also exhibit remarkably catalytic performance (Hmadeh et al., 2012; Zhao et al., 2017; Cheng et al., 2019; Liu et al., 2019; Shi et al., 2020; Hu et al., 2021; Xu et al., 2021).

In this study, we developed a high-performance non-enzymatic electrochemical glucose sensor by *in situ* growth of conductive copper MOF on copper foam (CF) using a simple one-step hydrothermal method. Under the optimal conditions of each parameter value, the prepared electrode Cu-MOF/CF showed excellent electrocatalytic activity for glucose oxidation at alkaline pH with high sensitivity, low detection limit, wide linear detection range, short response time, and excellent stability. Moreover, the results of the human serum test showed that the material had good practical application feasibility.

## Experimental Sections

### Physical Measurements

A Bruker D8 Advance X-ray diffractometer equipped with Cu-Kα radiation (λ = 1.5418 Å) was used to collect powder X-ray diffraction (PXRD) data at room temperature. Calculated PXRD patterns were generated using Mercury 3.0 (Zhao et al., 2015; Su et al., 2017). Scanning electron microscopy (SEM) images were obtained on a Hitachi SU8010 SEM at 15 kV with energy-dispersive X-ray (EDX). Transmission electron microscopy (TEM) measurements were carried out on a Bruker EMX 10/12. X-ray photoelectron spectroscopy (XPS) was performed on Thermo ESCALAB 250XI with aluminum-Kα radiation.

### Materials and Syntheses

All starting materials were commercially available and were used without further purification. Sodium hydroxide (NaOH), copper (II) acetate monohydrate (Cu(CH_3_COO)_2_·H_2_O), glucose (Glu), sodium chloride (NaCl), ascorbic acid (AA), lactose (Lac), uric acid (UA), fructose (Fru), and dopamine (DA) were purchased from Aladdin Ltd. (Shanghai, China). Human serum samples were purchased from Nanjing Bonason Biological Technology Co., Ltd. (The experiment complies with relevant ethical principles and participants agreed and knew the content of the experiment.) All reagents were used as received without further purification. Water used in this experiment was deionized water. 2,3,6,7,10,11-hexahydroxytriphenylene (HHTP) is prepared according to the literature method (Hmadeh et al., 2012).

**SCHEME 1:**
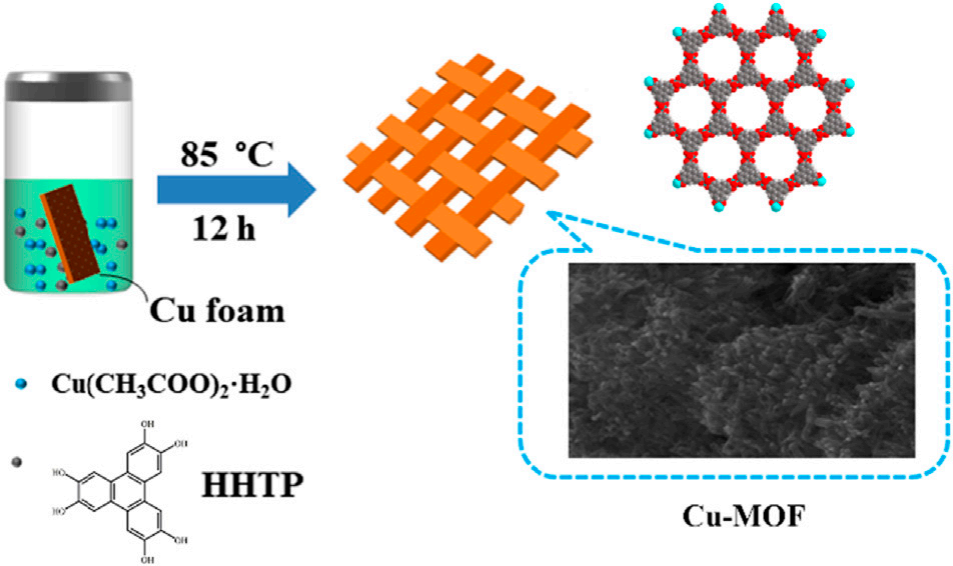
Structure and synthetic process of Cu-MOF.

**Preparation of Cu-MOF/CF**. Cu-MOF/CF was prepared using a facile one-step hydrothermal method (Scheme 1). CF was pre-cleaned by ultrasonicating sequentially in acetone, ethanol, and water for 15 min. HHTP (0.03 mmol, 7 mg) and Cu(CH_3_COO)_2_⋅H_2_O (0.08 mmol, 10 mg) were dissolved in H_2_O (4 ml). The mixture and the pretreated CF were placed in a 20-ml glass bottle and heated at 85°C for 12 h.

### Electrochemical Measurements

A CHI 660E (Chenhua, Shanghai) electrochemical workstation was used to conduct all electrochemical measurements at room temperature. The test was conducted using a three-electrode system: a platinum wire was used as the counter electrode, a mercuric oxide electrode (Hg/HgO) was used as the reference electrode, and the as-prepared Cu-MOF/CF was used as the working electrode. In addition, the geometric area of working electrodes was controlled at 0.2 × 0.2 cm^−2^ in all tests (Supplementary Scheme S1).

## Result and Discussion

### Material Characterization

**FIGURE 1:**
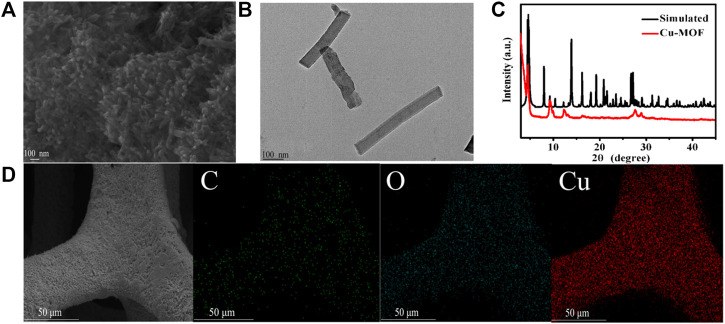
**(A)** SEM image of Cu-MOF/CF. **(B)** TEM figure of Cu-MOF. **(C)** PXRD pattern of the as-synthesized Cu-MOF. **(D)** SEM and EDX elemental mapping images of C, O, and Cu for Cu-MOF/CF.

**FIGURE 2:**
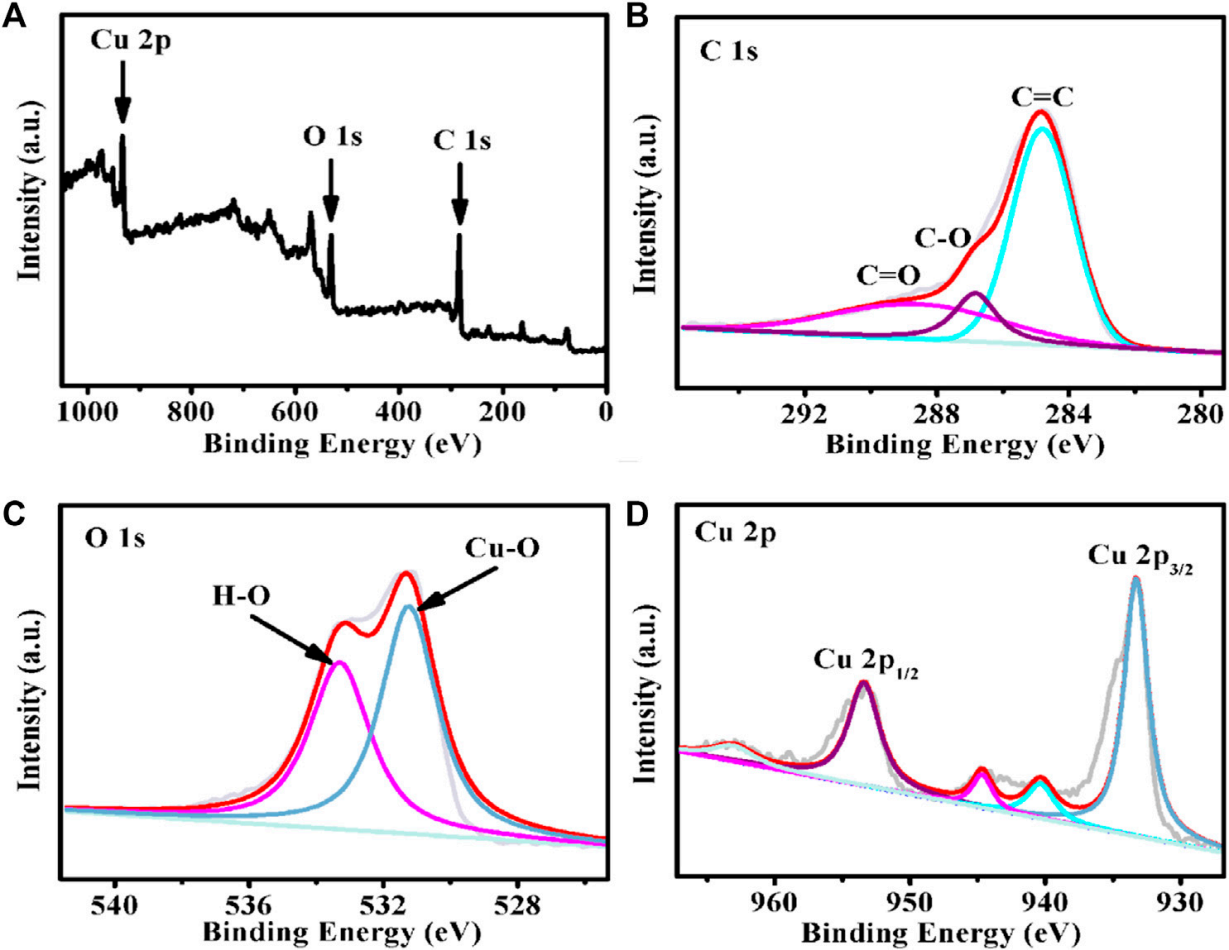
XPS spectrum for the Cu-MOF: **(A)** survey spectrum **(B)** C 1s, **(C)** O 1s, and **(D)** Cu 2p.

The SEM image of Cu-MOF/CF (Figure 1A) and TEM image for sample obtained by sonicating Cu-MOF/CF (Figure 1B) clearly show the rod-like morphology of Cu-MOF. The Cu-MOF nanorod arrays are completely and uniformly covered on the CF (Figure 1D). The powder X-ray diffraction (PXRD) spectra can be used to confirm the structural identity and phase purity of the synthesized materials. Because the intensity of the diffraction peak of PXRD of Cu-MOF/CF is too weak (Supplementary Figure S1), the PXRD of the Cu-MOF powder collected from the reaction system was used to further confirm structural identity and phase purity of the synthetic materials (Figure 1C). The significant peaks at 2θ = 4.6°, 9.4°, 12.4°, and 16.39° correspond to (100), (200), (130), and (201) planes, respectively (Ko et al., 2018; Hoppe et al., 2018; Qiao et al., 2020). The visible peaks at 2θ = 27.78° could be attributed to the (002) plane (Hmadeh et al., 2012; Li et al., 2019; Wu et al., 2019). The XRD spectrum shows that Cu-MOF has been successfully synthesized. The SEM image of Cu-MOF/CF and the corresponding energy-dispersive X-ray (EDX) element mapping image demonstrate the presence and homogeneous distributions on the bare CF of C, O, and Cu elements (Figure 1D). The corresponding energy-dispersive spectroscopy (EDS) further proves the presence of C, O, and Cu elements on the bare CF (Supplementary Figure S2). The elemental states and composition of Cu-MOF were studied by XPS. The full spectrum of XPS reveals the existence of C, O, and Cu elements in Cu-MOF, which corresponds with EDX (Figure 2A). The XPS peaks of C 1s can be observed at 283.98, and 287.72 eV (Figure 2B), which are composed of C–O and C–C, respectively (Jin et al., 2006; Yan et al., 2010; Tian et al., 2017). In the O 1s spectrum (Figure 2C), there are two O species, namely, O–Cu at 530.42 eV and O–H at 532.48 eV (Yang et al., 2020). As shown in Figure 2D, the main peaks at 932.47 and 952.58 eV in the Cu 2p spectrum are assigned to Cu 2p_3/2_ and Cu 2p_1/2_ spin-orbit states, respectively, which indicates the Cu^2+^ state in the Cu-MOF (He et al., 2016). The satellite peaks of Cu^2+^ can also be observed at 939.51, 943.84, and 962.24 eV (Liu et al., 2013; Tian et al., 2017).

### Electrochemical Characterizations

**FIGURE 3:**
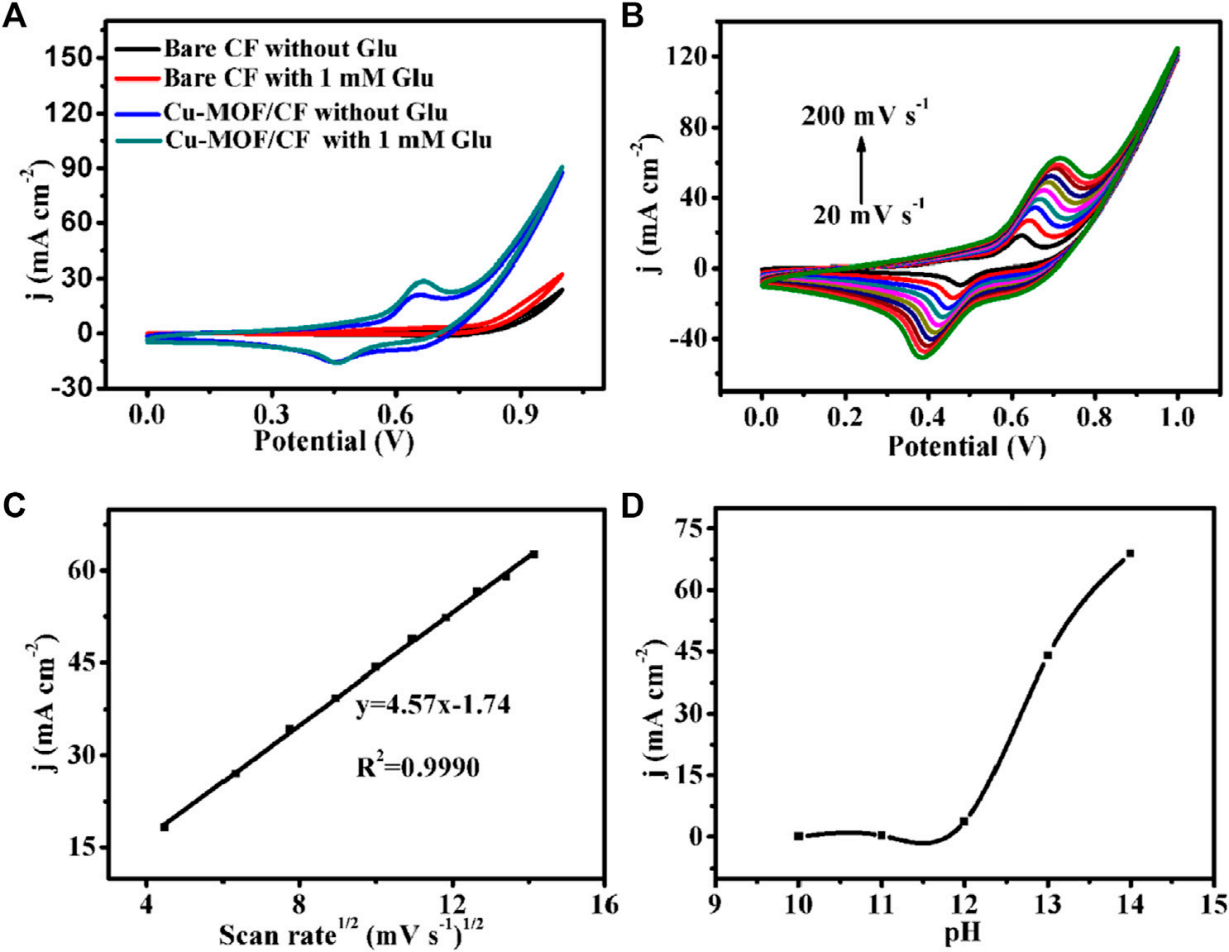
**(A)** CVs of bare CF and Cu-MOF/CF in 0.1 M NaOH in the absence and presence of 1 mM Glu (scan rate: 50 mV s^−1^). **(B)** CVs for Cu-MOF/CF in 1 mM Glu at scan rates from 20 to 200 mV s^−1^. **(C)** Corresponding plot of current density *vs*. the square root of the scan rate. **(D)** Plot of anodic peak current density *vs* pH for the Cu-MOF/CF electrode in the presence of 1 mM glucose at 0.65 V (scan rate, 50 mV s^−1^).

In order to investigate the electrocatalytic performance of the as-prepared catalyst for glucose oxidation under alkaline pH conditions, electrochemical tests were carried out using a typical three-electrode setup in 0.1 M NaOH. The cyclic voltammograms (CVs) were performed at a scan rate of 50 mV s^−1^ within the potential range of 0–1 V (Figure 3A). The bare CF did not show any visible redox peak in the absence or presence of glucose, indicating no catalytic activity of the bare CF for glucose. Cu-MOF/CF displayed a reversible oxidation and reduction wave, confirming redox activity and providing the possibility for electrochemical sensing. A significant enhancement of the oxidation current was observed on the addition of 1 mM glucose solution into the electrolyte, indicating that the Cu-MOF/CF has electrocatalytic activity for glucose oxidation. The possible corresponding reaction process of the as-prepared sensor for glucose oxidation can be predicted as follows (Lan et al., 2020; Pa et al., 2020; Zheng et al., 2020):

$$\text{Cu}\left(\text{II}\right)-\text{MOF/CF}+{\text{OH}}^{-}\to \text{Cu}\left(\text{III}\right)-{\text{MOF/CF}+\text{e}}^{-}+{\text{H}}_{2}\text{O,}$$ (1)

Cu(III)-MOF/CF+Glucose→Cu(II)-MOF/CF+Gluconolactone, (2)

$${\text{Gluconolactone }+\text{H}}_{2}\text{O}\to {\text{H}}^{+}+\text{Gluconate}\text{.}$$ (3)

In order to study the electrochemical process of glucose, the CVs of Cu-MOF/CF in 0.1 M NaOH with 1 mM glucose were recorded at different scan rates from 20 mV s^−1^ to 200 mV s^−1^ (Figure 3B). As depicted in Figure 3C, the oxidation peak and reduction peak current densities increase with increasing scan rates. The oxidation peak currents are directly proportional to the square root of the scan rate in the rage of 20–200 mV, suggesting that the electrochemical oxidation process of glucose at the working electrode is diffuse-controlled (Zhu et al., 2018). Meanwhile, the oxidation peak moves to a more positive potential, and the reduction peak moves to a more negative potential (Karikalan et al., 2016). The electrochemical response of Cu-MOF/CF to 1 mM glucose under different pH conditions was evaluated in an electrolyte containing 1 mM glucose at pH values of 10–14 (Figure 3D). The peak current density of the oxidation peak increases with the increase in pH value. However, the oxygen evolution reaction at pH 14 can cause a sharp increase in the catalytic current density which can interfere with glucose detection. Therefore, pH 13 was chosen as the optimal pH value for follow-up experiments in this study.

**FIGURE 4:**
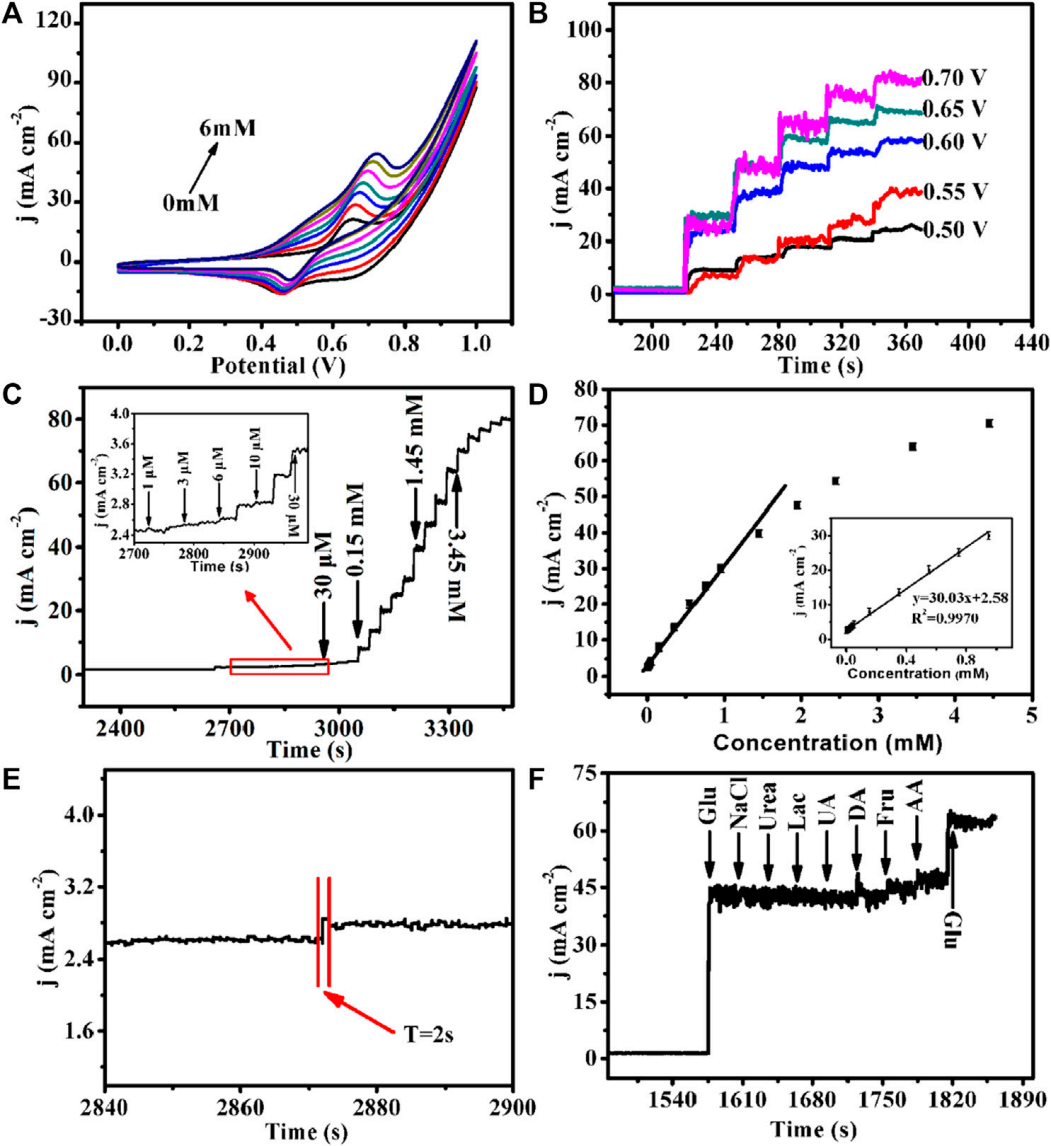
**(A)** Cu-MOF/CF in 0.1 M NaOH with the presence of varied Glu concentrations: 0, 1, 2, 3, 4, 5, and 6 mM (from inner to outer) at a scan rate of 50 mVs^−1^. **(B)** Amperometric responses of the Cu-MOF/CF electrode at different potentials (from 0.50 to 0.7 V) with continuous addition of 1 mM Glu in 0.1 M NaOH. **(C)** Amperometric response of Cu-MOF/CF with successive addition of Glu in 0.1 M NaOH (inset: the current response of the electrode toward the addition of Glu from 1 μΜ to 30 μM) and **(D)** Corresponding calibration curve of Cu-MOF/CF electrode to successive additions of Glu at 0.65 V in 0.1 M NaOH. **(E)** Amplification of the Cu-MOF/CF amperometric response curve. **(F)** Amperometric response of the Cu-MOF/CF electrode toward the addition of Glu (1 mM) with various interfering species (1 mM) in 0.1 M NaOH.

Moreover, the CV curves of Cu-MOF/CF with glucose concentration from 0 to 6 mM were tested to further study the electrochemical performance. As shown in Figure 4A, when the concentration of glucose increases, the anodic current density increases and the anodic oxidation peak shifts to the corrected position, demonstrating superior electrocatalytic activity. In order to find the optimal voltage for the detection, at a fixed scanning rate, glucose (1 mM) was successively added to the NaOH (0.1 M) electrolyte at voltages of +0.5, +0.55, +0.6, +0.65, and +0.7 V (Figure 4B). The five potentials exhibit similar current responses. The current density increases with the increase in the working potential and reaches the maximum at +0.7 V. Compared with +0.7 V, +0.65 V exhibits relatively small noise on the current, so it was chosen as the optimal voltage for further tests. Under the optimal voltage (+0.65 V), glucose solutions of different concentrations and volumes to the NaOH (0.1 M) electrolyte were used to make the glucose concentration in the electrolyte continuously change to obtain the final current–time diagram (Figure 4C). The amperometric responses of Cu-MOF/CF on different concentrations of glucose were recorded by successively adding different concentrations and volumes of glucose to the electrolyte, with concentrations ranging from 1 uM to 3.45 mM under constant stirring (Figure 4C). The anode current increases obviously with the addition of glucose. Figure 4D illustrates the relationship between glucose concentration and current response signal. The current density and glucose concentration have a linear relationship within the range of 0.001–0.95 mM. The corresponding linear equation is j (mA cm^−2^) = 30.03c + 2.58 (*R* ^2^ = 0.997), and the sensitivity was 30,030 mA μM^−1^ cm^−2^. According to the 3-fold signal/noise value (S/N = 3) (Zhou et al., 2020), the limit of detection (LOD) was 0.076 μM. Meanwhile, it takes only 2s to reach a steady-state current density from one concentration to an adjacent high concentration, which reveals that the prepared Cu-MOF/CF electrode has a fast ampere response to glucose (Figure 4E). All the results show that Cu-MOF/CF exhibits better electrocatalytic performance with rather high sensitivity, low LOD, and wide detection range than most reported non-enzymatic sensors (Supplementary Table S1) and provided a promising prospect for MOF-based materials as glucose sensors. For biological systems, glucose often coexists with biological molecules, such as ascorbic acid, urea, uric acid, NaCl, and dopamine, and some other sugar molecules such as lactose and fructose which may disturb the detection of glucose. So, the anti-interference tests are essential. In order to study the selectivity of the prepared Cu-MOF/CF, we conducted anti-interference tests by recording the current responses with adding the aforementioned interferences in the electrolyte. As shown in Figure 4F, a salient signal appeared on adding glucose, but with the addition of other interfering substances (0.1 mM lactose (Lac), 0.1 mM fructose (Fru), 0.1 mM ascorbic acid (AA), 1 mM urea (UR), 0.1 mM uric acid (UA), 1 mM NaCl, and 0.1 mM dopamine (DA)), no obvious signal could be observed. Considering that the concentration of glucose in the physiological environment is more than 30 times that of interfering substances, the Cu-MOF/CF with good selectivity has strong practical application feasibility.

**FIGURE 5:**
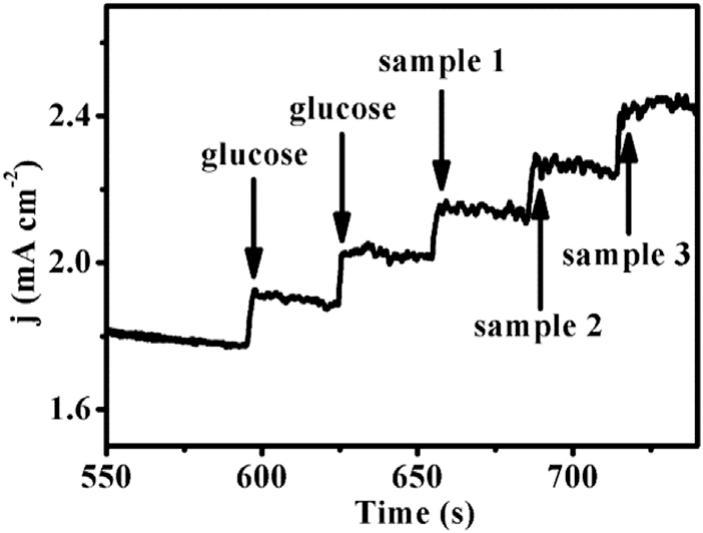
Amperometric response of the Cu-MOF/CF with addition of glucose and three serum samples (glucose: 5 mM, potential: 0.65 V, solution: 0.1 M NaOH).

In order to verify the practical application feasibility of the sensor, Cu-MOF/CF was used to determine the glucose concentration in human serum samples at 0.65 V. Three blood samples were tested. The ampere–current responses of the serum sample are shown in Figure 5. And the measured values were almost on a par with the value from the commercial glucometer. The standard deviations and the relative standard deviation (RSD) were less than 0.22 and 3.44%, respectively (Table 1). The aforementioned results show that this method is accurate and reliable. Therefore, the sensor can provide an effective method for the determination of glucose in practical samples.

**TABLE 1:** Sensor in this study and the blood glucose meter test on serum samples (n = 3).

Sample	Measured by glucometer (mM)	Determined by the sensor (mM)	SD	RSD (%)
1	4.8	5.09	0.16	3.25
2	5.2	5.13	0.08	1.48
3	6.4	6.64	0.22	3.44

All the concentration tests and RSD calculations are of three independent measurements. SD, standard deviation; RSD, relative standard deviations.

**FIGURE 6:**
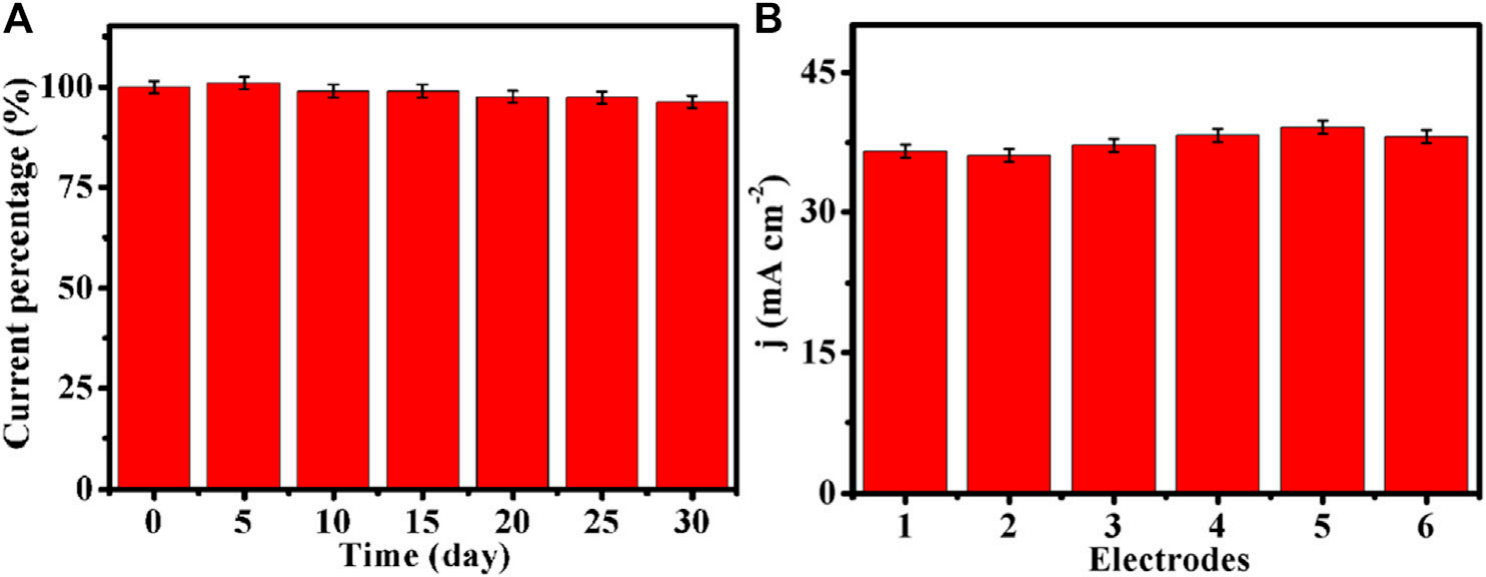
**(A)** Stability of Cu-MOF/CF to 1 mM glucose tested every 7 days by cyclic voltammetry in 1 month. **(B)** Reproducibility of six biosensors toward 1 mM glucose in 0.1 M NaOH.

Stability is also a critical parameter for practical applications of sensors. The long-term stability experiments of Cu-MOF/CF were carried out every 7 days by cyclic voltammetry in 1 month. As shown in Figure 6A, the current density can retain 96% of initial catalytic activity after a month of stability tests, indicating good stability of Cu-MOF/CF. In order to investigate the reproducibility of the electrodes, six Cu-MOF/CF electrodes were prepared separately under the same conditions. Glucose oxidation was tested at six independent sensors (Figure 6B). The SD and RSD of the six electrodes to 1 mM glucose current were about 1.15 and 3%, respectively (Supplementary Table S2), indicating noticeable reproducibility.

## Conclusion

In summary, without using any polymer binder, the conductive Cu-MOF was successfully grown on the CF by the one-step hydrothermal method and could act as an effective catalyst electrode for the electrochemical oxidation of glucose under alkaline conditions. As a high-efficiency electrochemical sensor for non-enzymatic glucose detection, the material exhibits excellent catalyst performance with a short response time (2 s), a wide detection range (0.001–0.95 mM), a low detection limit (0.076 μM, S/N = 3), strong sensitivity (30,030 mA μM^−1^ cm^−2^), and good stability and repeatability. This study not only demonstrates excellent performance of MOF-based materials and their potential as a promising platform for the fabrication of highly sensitive electrochemical biosensors but also provides an attractive, cost-effective, easily prepared electrode material for efficient glucose detection.

## Data Availability

The original contributions presented in the study are included in the article/Supplementary Material; further inquiries can be directed to the corresponding authors.
