# Supplementary material for: Cu-Based Conductive MOF Grown in situ on Cu Foam as a Highly Selective and Stable Non-Enzymatic Glucose Sensor
Source: Front Chem. 2021 Nov 29;9:786970. doi: 10.3389/fchem.2021.786970 (PMC8666423; doi:10.3389/fchem.2021.786970)
Supplement: Supplementary file 1 [file DataSheet1.docx]

Supplementary Material for

**Cu-based Conductive MOF in-situ Grown on Cu Foam as a Highly Selective and Stable Nonenzymatic Glucose Sensor**

Qin Hu^1§^, Jie Qin^2§^, Xiao-Feng Wang^3^, Guang-Ying Ran^1^, Qiang Wang^1^, Guang-Xiang Liu^3*^, Jian-Ping Ma^4*^, Jing-Yuan Ge^5*^ and Hai-Ying Wang^1,3*^

*^1^College of Chemistry and Materials Science, Sichuan Normal University, Chengdu 610066, P. R. China*

*^2^School of Life Sciences and Medicine, Shandong University of Technology, Zibo 250000, P. R. China*

*^3^School of Environmental Science, Nanjing Xiaozhuang University, Nanjing 211171, P. R. China*

*^4^School of Chemistry, Chemical Engineering and Materials Science, Shandong Normal University, Jinan 250014, P. R. China*

*^5^College of Chemistry & Materials Engineering, Wenzhou University, Wenzhou 325035, P. R. China*

.**Contents**

**Table S1.** Comparison of the performance of the **Cu-MOF/CF** with other reported MOFs-based non-enzymatic glucose sensors.

**Supplementary Scheme 1.** Device for electrochemical glucose sensing.

**Supplementary Figure 1.** XRD spectra of Cu-MOF, **Cu-MOF/CF** and CF.

**Supplementary Figure 2.** EDS spectra of **Cu-MOF/CF**.

**Table S2** The reproducibility of electrodes.

**Supplementary References**

**Table S1.** Comparison of the performance of the **Cu-MOF/CF** with other reported MOFs-based non-enzymatic glucose sensors.

| **Electrode** | **Linear range**  **(mM)** | **Sensitivity**  **(μA mM^−1^ cm^−2^)** | **LOD**  **(μM)** | **Ref.** |
| --- | --- | --- | --- | --- |
| Cu-MOF | 10-3500 | 89 | 2.40 | (1) |
| Cu/Cu_2_O nanocomposites | Up to 40 | 1434.12 | 1.60 | (2) |
| Cu@HHNs/GCE | 0.0005-3 | 1594.2 | 1.97 | (3) |
| Cu_3_P NW/CF | 0.005-1 | – | 0.32 | (4) |
| Ag@ZIF-67/GCE | 0.002-1 | 379 | 0.66 | (5) |
| Cu@porous carbon matrix | 0.001-0.3 | 10100 | 0.60 | (6) |
| UiO-67@Ni-MOF/GCE | 0.005-0.55  0.55-3.9 | 203.4 | 0.98 | (7) |
| Ni-MOF/Ni/NiO/C GCE | 0.004-5.664 | 364.75 | 0.80 | (8) |
| Cu-on-ZIF-8 | 0-700 | 412 | 2.76 | (9) |
| **Cu-MOF/CF** | 0.001-1.45 | 30030 | 0.076 | This work |

**
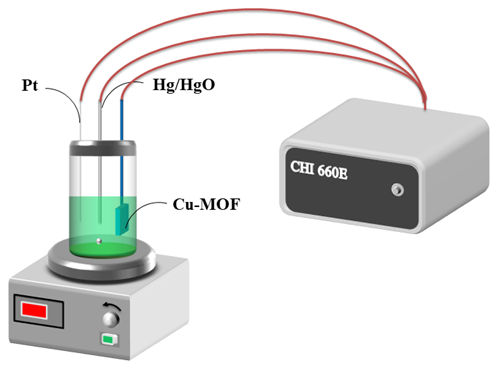
**

**Supplementary Scheme 1.** Device for electrochemical glucose sensing.

**

**

**Supplementary Figure 1.** XRD spectra for Cu-MOF, **Cu-MOF/CF** and CF.


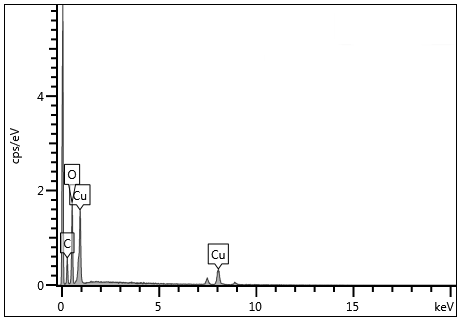


**Supplementary Figure 2.** EDS spectra of **Cu-MOF/CF**.

**Table S2** The reproducibility of electrodes.

| **Electrode** | **j (mA cm^−2^)** | **$\overline{\text{j}}$(mA cm^−2^)** | **SD** | **RSD (%)**  **(n=6)** |
| --- | --- | --- | --- | --- |
| 1 | 36.54 | 37.55 | 1.15 | 3.06 |
| 2 | 36.07 |  |  |  |
| 3 | 37.19 |  |  |  |
| 4 | 38.25 |  |  |  |
| 5 | 39.12 |  |  |  |
| 6 | 38.12 |  |  |  |

The RSD calculations are of six independent measurements.

**j:** Current density **$\overline{\text{j}}$:** Average current density

**SD**: Standard deviations **RSD**: Relative standard deviations

**Supplementary References**

1. Sun, Y., Li, Y., Wang, N., Xu, Q. Q., Xu, L., & Lin, M. (2018). Copper-based Metal-organic Framework for Non-enzymatic Electrochemical Detection of Glucose, *Electroanalysis* 30, 474-478.
2. Cheng, X., Zhang, J., Chang, H., Nie, F., Luo, L., Feng, X. (2016). High performance Cu/Cu_2_O nanohybrid electrocatalyst for nonenzymatic glucose detection. *J. Mater. Chem. B*. 4, 4652-4656.
3. Zhu, Q., Hu, S., Zhang, L., Li, Y., Carraro, C., Maboudian, R., Wei, W., Liu, A., Zhang, Y., Liu, S. Reconstructing hydrophobic ZIF-8 crystal into hydrophilic hierarchically-porous nanoflowers as catalyst carrier for nonenzymatic glucose sensing. *Sens. Actuators B Chem*. 313, 128031.
4. Xie, L., Asiri, A. M., & Sun, X. (2017). Monolithically integrated copper phosphide nanowire: An efficient electrocatalyst for sensitive and selective nonenzymatic glucose detection, *Sens. Actuators B Chem*. 244, 11-16.
5. Meng, W., Wen, Y., Dai, L., He, Z. X., Wang, L. (2018). A novel electrochemical sensor for glucose detection based on Ag@ZIF-67 nanocomposite. *Sens. Actuators B Chem*.260, 852-860.
6. Zhang, X., Luo, J. S., Tang, P. Y., Morante, J. R., Arbiol, J., Xu, C. L., Li, Q. F., Fransaer, J. (2018). Ultrasensitive binder-free glucose sensors based on the pyrolysis of in situ grown Cu MOF. *Sens. Actuators B Chem*. **2018**, 254, 272-281.
7. Lu, M. X., Deng, Y. J., Li, Y. C., Li, T. B., Xu, J., Chen, S. W., Wang, J. Y. (2020). Core-shell MOF@MOF composites for sensitive nonenzymatic glucose sensing in human serum. *Anal. Chim. Acta.* 1110, 35-43.
8. Shu, Y., Yan, Y., Chen, J. Y., Xu, Q., Pang, H., Hu, X. Y. (2017). Ni and NiO nanoparticles decorated metal-organic framework nanosheets: facile synthesis and high-performance nonenzymatic glucose detection in human serum. *ACS Appl. Mater. Inter*. 9, 22342-22349.
9. Shi, L., Xiang, Z., Liu, T., Zhao, H., Lan, M. (2016). Encapsulating Cu nanoparticles into metal-organic frameworks for nonenzymatic glucose sensing, *Sens. Actuators B Chem*. 227, 583-590.
